# Elucidating genetic variability between randomly bred domestic cats and Persian domestic cats from different geographical locations using microsatellite markers

**DOI:** 10.1002/vms3.70004

**Published:** 2024-10-18

**Authors:** Shirin Mahmoodi, Ali Hojabr Rajeoni, Mehrshad Zeinolabedini, Arash Javanmard, Mohammad Hossein Banabazi

**Affiliations:** ^1^ National Center of Genetic Resources Agricultural Research Education and Extension Organization (AREEO) Tehran Iran; ^2^ Department of Microbiology and Immunology Faculty of Veterinary Medicine University of Tehran Tehran Iran; ^3^ Department of Genomic Agricultural and Biotechnological Research Institute of Iran (ABRII) Agricultural Research Education and Extension Organization (AREEO) Karaj Iran; ^4^ Department of Animal Science Faculty of Agriculture, University of Tabriz Tabriz Iran; ^5^ Department of Animal Breeding and Genetics (HGEN) Centre for Veterinary Medicine and Animal Science (VHC) Swedish University of Agricultural Sciences (SLU) Uppsala Sweden; ^6^ Department of Biotechnology Animal Science Research Institute of IRAN (ASRI), Agricultural Research, Education & Extension Organization (AREEO) Karaj Iran

**Keywords:** domestic cat, genetic variability, inbreeding, microsatellites

## Abstract

**Background:**

The domestic cat (*Felis catus*) is a newly evolved species in the family Felidae that has developed some great features among mammals. It is critical to conserve these species and prevent inbreeding from reducing their genetic diversity by understanding their genetic relationships and applying the information to breeding management. The diverse population was an excellent choice for studying genetic diversity and inbreeding phenomena.

**Objectives:**

To conduct this research, 128 individuals from 8 populations, including Azerbaijan, Persian, Ahar, Uermia, Tehran, Karaj, Turkish and Shop cat (both genders), were randomly selected from different geographical regions. We selected eight STR markers with different chromosomal locations based on polymorphism and observed allele numbers in the next step. DNA extraction was performed using tail hair root, PCR and electrophoresis, and gel staining was performed according to routine laboratory protocol. For statistical analysis, CONVERT versions POPGENE, ARLEQUIN GenAlEx and R script analysis.

**Results:**

Remarkably, our results showed that 23 alleles were identified in 128 samples. The highest number of alleles belonged to the FCa096 locus (eight alleles) in the Persian population, followed by FCa045 (seven alleles) in the Persian and Ahar populations. Another new finding is that the lowest number of alleles belonged to the 35 and FCa77 locus (two alleles). In addition, pairwise differentiation between and within populations was examined using the genetic distance index. Overall, the results showed that the degree of differentiation within the population is high in the Turkish population compared to other population groups and lower in the Azerbaijan population. In addition, principal component discriminant analysis‐based analysis based on the ADAGENET package shows the distribution of samples by geographical location. The results show that genetic mixing between populations is high.

**Conclusions:**

On this basis, we conclude that randomly bred domestic cats have a higher level of diversity than Persian domestic cats. This is an interesting topic for future work.

## Graphical abstract

This research surveyed 128 cats from 8 populations, including Azerbaijan, Persian, Ahar, Uremia, Tehran, Karaj, Turkish and pet shop. Eight STR markers were selected with different chromosomal locations based on polymorphism. DNA extraction using tail hair root and PCR was performed according to laboratory protocol. Results showed Persian cat population exhibits the highest number of alleles belonged to the FCa096 locus (eight alleles) followed by FCa045. The degree of differentiation within the population is lower in the Azerbaijan population.

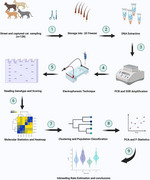

## 1 INTRODUCTION

The domestic cat (*Felis catus*) is a newly evolved species in the family Felidae that has developed some great features among mammals over the last 11 million years (Driscoll et al., 2009). The domestic cat is now the most common pet in Iranian families (Mohebali et al., 2022). Today, almost 50 cat breeds are recognized. Among these 50 breeds, 15 are main breeds, or breeds believed to have evolved naturally due to geographical isolation and adaptation to their habitat (Kurushima et al., 2013; Nilson et al., 2022). Generally, very little is known about the population genetics of cat breeds. The mating behaviour of street cats is an independent variable, and the choice of mating depends on the cat's desire (Phandee et al., 2016). The breeding of Persian cats is controlled by humans, unlike randomly bred domestic cats, so their genetic diversity and inbreeding situation are influenced by humans, which is different from street cats (Lipinski., 2008). Because wild cats and highly selective cat breeds are in different stages of domestication, randomly bred cats are in the middle. Persian cats, compared to randomly bred cats, likely reflect only the most recent epoch of migration and interbreeding (Pickrell & Reich, 2014). The genetic diversity of randomly bred cats reflects an uncertain and potentially changing historical period (Lyons et al., 2014). It is crucial to understand the genetic relationships between these species and use the information to manage breeding to preserve their genetic diversity and control inbreeding (Kurushima et al., 2013; Lipinski et al., 2008). A breeding programme to lessen inbreeding in captive animals is crucial for conservation genetics. There is a higher chance of extinction as a result of inbreeding, which lowers genetic variability, fertility and survival rates (Farquharson et al., 2021; Humble et al., 2023; Klimova et al., 2022; Urzi et al., 2021). A reduction in heterozygosity through inbreeding occurs when individuals related by descent mate. Genetic selection, breed management and population prevention of inbreeding are not currently addressed in any genetic study of native Iranian cats.

Microsatellites are PCR‐based markers consisting of short DNA segments that repeat forming series with length of up100 nt (1‐ 6 bp). Microsatellites are an excellent tool for assessing genetic diversity between closely related populations due to their high mutation rate, estimated at 10^2^–10^6^ per generation (Abdul‐Muneer et al., 2014; Vieira et al., 2016; Wang et al., 2021; Wenne, 2023). In the cat genome (2*N* = 38), the most common microsatellite repeats were (CA)*n* or (GT)*n*, approximately every 40 kb apart (Menotti‐Raymond et al., 2008). For example, microsatellites with F_ST_ can detect the genetic mismatch of subpopulations and measure and characterize different populations’ genetic diversity (Hyun et al., 2021; Wright, 1978). Additionally, structural software can be used to identify genetic admixture between subpopulations via microsatellites (Funk et al., 2020; Kaeuffer et al., 2007; Porras‐Hurtado et al., 2013; Pritchard et al., 2000). Therefore, these markers and techniques are widely used in the study of subpopulations or species conservation. However, microsatellites have certain limitations, such as the presence of null alleles, and it is important to consider these limitations. The population’*s* high level of diversity made it a great option for research into inbreeding and genetic diversity (Barrett et al., 2022; Coltman & Slate, 2003; Lyu et al., 2023; Sánchez‐Velásquez et al., 2022).

This motivation led to the overall goal of current work, which was to determine whether microsatellites could be used to elucidate genetic variability in Persian and domestic cats.

## 2 MATERIALS AND METHODS

### 2.1 Animals and sampling

**FIGURE 1:**
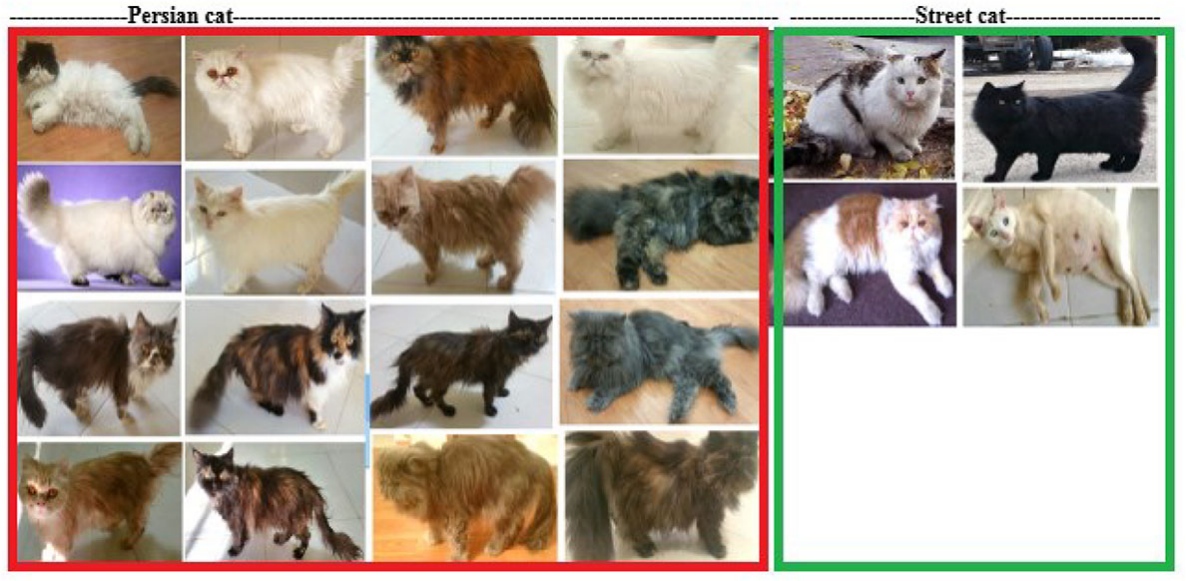
A detailed description of the cat breeds examined and the different affiliations of Persian and street cats. Red square is representative of Persian cats and green one highlighting street cats.

To conduct this research, a total of 128 individuals from 8 populations, including Azerbaijan, Persian, Ahar, Uermia, Tehran, Karaj, Turkish and Shop cat (both sexes), were randomly selected from different geographic regions, Figure 1 provides a detailed description of the studied cat breeds and different appurtenance of Persian and street cats.

Extensive documentation was kept about the location of the sampling, the identity of the cat and its owner. A group of cats were selected from pet stores and shops selling Persian cats in cities in Iran. These cats were chosen for selective mating by the owners based on their colour, appearance, body morphology and buyer criteria. They were marketable and mated mainly with a few and well‐known privileged male.

### 2.2 Hair sampling and DNA extraction

After the required samples were selected and cut, the caudal hair root was stored at 4°C. Individual hairs were randomly removed with tweezers, and the presence of a root mark was visually checked. To avoid cross‐contamination, instruments were carefully cleaned and dried between samples. A sterile centrifuge tube was used for each hair. For some samples, whole individual hairs were added unchanged to the digestion buffer. To ensure no roots were present, approximately 1 mm of both hair tips were cut off from some samples, and the remaining portion of the hair shaft was placed in a digestion buffer. A digestion buffer consisting of 10 mM Tris–HCl, 1 mM EDTA, 0.5 mg proteinase K (Roche Applied Science) and 0.2 M dithiothreitol (Promega Corporation) was dissolved in 200 µL for 24 h, for each hair sample), then be sured about completely digested process. Ten percent SDS and 0.5 M NaCl (Teknova, Hollister) were used. After 1 h of digestion, all samples were shaken for 30 s to break up the hair mechanically. After4 h, visual inspection revealed that the lighter‐pigmented and thinner hairs were completely digested. All samples were still digested for a full day to ensure complete dissolution. After digestion, samples were centrifuged at 15,000 *g* for 2 min, refrigerated for another night, and heated to 95°C for 20 min. After transferring the supernatant to sterile tubes, the pellet was discarded. The typical NaCl–ethanol method was used to precipitate the DNA. Before analysis, the DNA pellets were frozen at 80°C and reconstituted in 50 µL ddH_2_O.

### 2.3 PCR assay and electrophoresis condition

Based on the polymorphism and observed allele number described in the previous literature, we selected eight STR markers with different chromosomal positions accordingly (Table 1). Table 1 addresses an overview of eight STR markers in the present study.

**TABLE 1:** An overview of eight STR markers in the present study.

Loci	Pattern	Sequence (5 to 3 dimensions)	PIC	Motif	Chr.	PCR Size
FCa8	Polymorphic primers	ACTGTAAATTTCTGAGCTGGCC TGACAGACTGTTCTGGGTATGG	0.98	(CA)_n_	A_1_	122–148
Fca096		CACGCCAAACTCTATGCTGA CAATGTGCCGTCCAAGAA	0.76	(CT)_n_	A_2_	184–224
FCa35		CTTGCCTCTGAAAATGTAAATG AAACGTAGGTGGGGTTAGTGG	0.73	(GT)_n_	D_2_	136–150
FCa045		TGAAGAAAAGAATCAGGTGTG TATGAGCATCTCTGTGTTCGT	0.85	(CA)_n_	A_1_	146–160
FCa77		GGCACCTATAACTACCAGTGTGA ATCTGGGGAAATAAATTTTGG	0.58	(TG)_n_	C_2_	143–155
FCa013	Monomorphic primers	GGGAGGGAGCCTATTTAAGA AAGATTGGATGCTTACCG	0.40	–	B13	160–170
Fc018		GCCGGATATGGGTTTTTCAT ATCCCAAAAACAACAGACAACA	0.55	–	X	221–225
FCA05		CCTAAGGAAACAGTAATCCTGGC TGGCAGGGCACCAGGAT	0.68	–	E1	148–154

**Reference**: (Menotti‐Raymond et al., 2008).

A NanoDrop 2000/2000c spectrophotometer and gel monitoring tools were used to measure quantity and quality (Thermo Fisher Scientific). The PCR solution had a total volume of 15 µL and contained a master mix kit (Ampliqon), 7 µL Master Mix 2X, 1 pmol of each primer, 4 µL ddH_2_O and 1 µL genomic DNA. All these procedures were performed on ice.

The PCR programme was used in the PCR machine to amplify the STR loci of the Touchdown PCR‐specific programme, which was designed to amplify the locus and minimize nonspecific and starter bands simultaneously. Biometra has modelled the PCR machine for amplifying fragments.

A detailed Touchdown amplification protocol was performed in the following manner to reduce stutter band and genotyping errors (Seo et al., 2014; Trobajo‐Sanmartín et al., 2018): (a) initial denaturation (94°C–6 min); (b) 1 cycle of denaturation (94°C–45 s), annealing (66°C–50 s) and elongation (72°C–50 s); (c) 1 cycle of denaturation (94°C–45 s), annealing (64°C–50 s) and elongation (72°C–50 s); (d) 1 cycle of denaturation (94°C–45 s), annealing (62°C–50 s) and elongation (72°C–50 s); (f) 1 cycle of denaturation (94°C–45 s), annealing (60°C–50 s) and elongation (72°C–50 s); (g) 25 cycles of denaturation (94°C–4 s), annealing (58°C–50 s) and extension (72°C–50 s) and final extension at 72°C for 5 min.

The PCR products were identified using 6% Metaphor agarose gels stained with Safe dye in 1× TBE buffer. The 11‐line ladder (25–755  fragemets size bp) (Life Science Company) was used to estimate allele sizes.

#### 2.3.1 Statistical analysis

UVdoc software measures individual genotypes or allele sizes at each site in this assay. The packages CREAT version 1.1 (Coombs et al., 2008) and CONVERT version 1.31 (Glaubitz, 2004) were then employed to prepare the input files for every package. The molecular measurement indices were calculated, including genetic distance, allelic admixture, population structure, clustering, analysis of molecular variance, genotype and allele frequencies, Na, Ne, Ho, He, I, PIC and *F* statistics (*F*_IS_, *F*_IT_ and *F*_ST_). Among the numerous computer programmes and applications utilized is POPGENE version 1.31 (Yeh et al., 1998(B; ARLEQUIN version 3.5, 1999. GenAlEx version 6.5 (Peakall & Smouse, 2012) and 2.2 (Excoffier & Lischer, 2010; Jombart & Collins, 2015).

## 3 RESULTS

### 3.1 Allele frequencies within and between studied populations

Based on allele frequencies evidence, an allele with a size of 112–226 bp was obtained after eight studied polymerase chain reaction sites were amplified and showed polymorphism. A total of 128 samples yielded 23 allele discoveries. In the Persian population, the FCa096 locus (eight alleles) had the largest number, whereas the Ahar and Persian populations had seven alleles for FCa045. Locus FCa35 and FCa77 (two alleles) had the lowest number. The average number of alleles for each site is approximately 3.8. Among them, samples from Azerbaijan contained 16, Persian 21, Ahar 22, Uermia 20, Tehran 20, Karaj 20 and Shop cat 20 alleles. A total of two specific alleles belonging to the FCa096 locus (alleles 173 and 193 bp) in the Ahar and Persian populations were found in the samples. The frequency of each allele was 0.042 and 0.02, respectively. The number of alleles for the Ahar population was higher than that of other populations, and the number of alleles for the Turkish population was lower than that of others. The average number of real and effective alleles was 3.8 and 2.56, respectively, and the allelic range was between 2 and 8. Figure 2A addressed molecular statistical description for five polymorphic loci in this study and Figure 2B indicates a snapshot of the overall observed allele number per studied population. A molecular statistical description for five polymorphic loci used in this study is depicted in Figure 1B.

FIGURE 2(a) Molecular statistical description for five loci in studied populations and (b) Snapshot of overall observed allele number per studied population.

**Figure:**
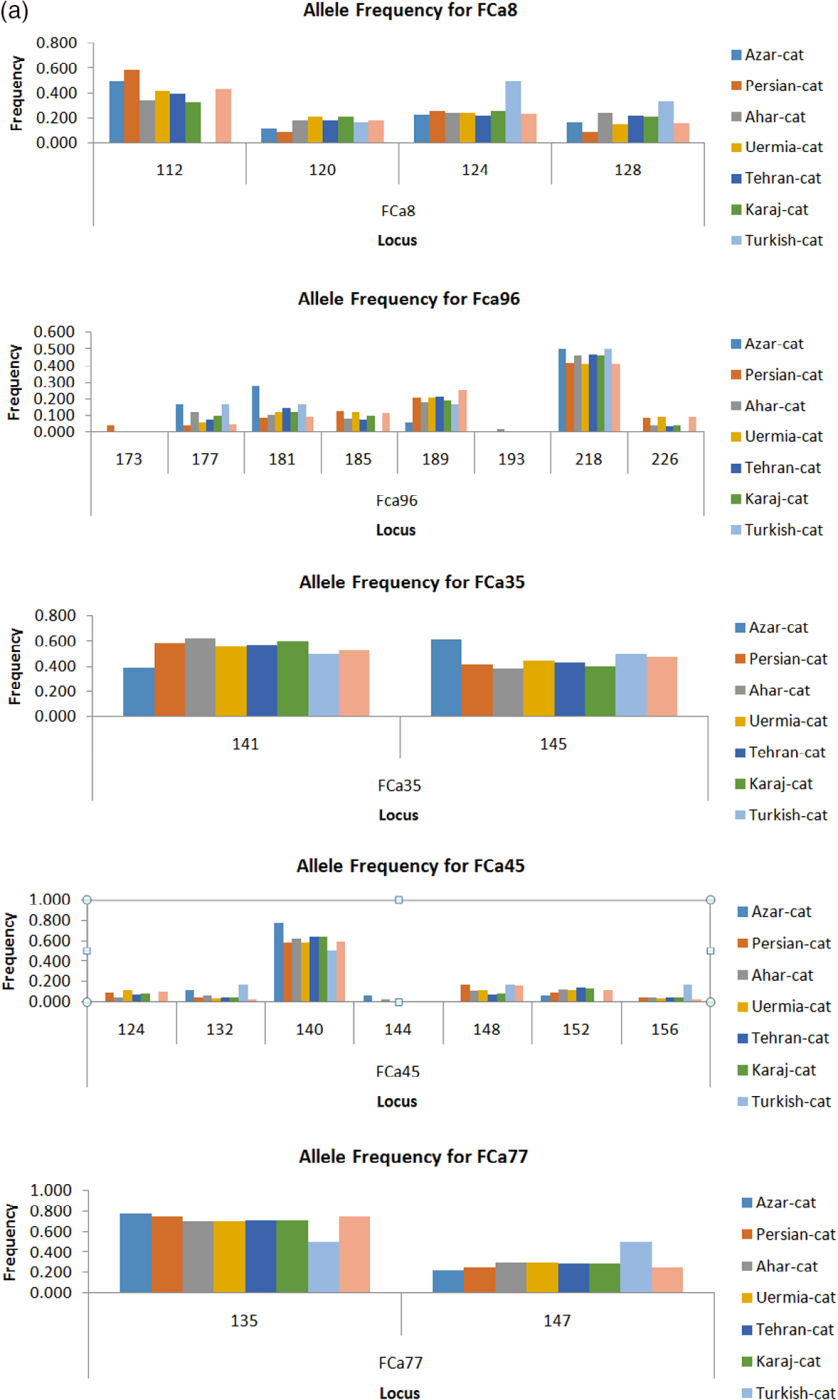

**Figure:**
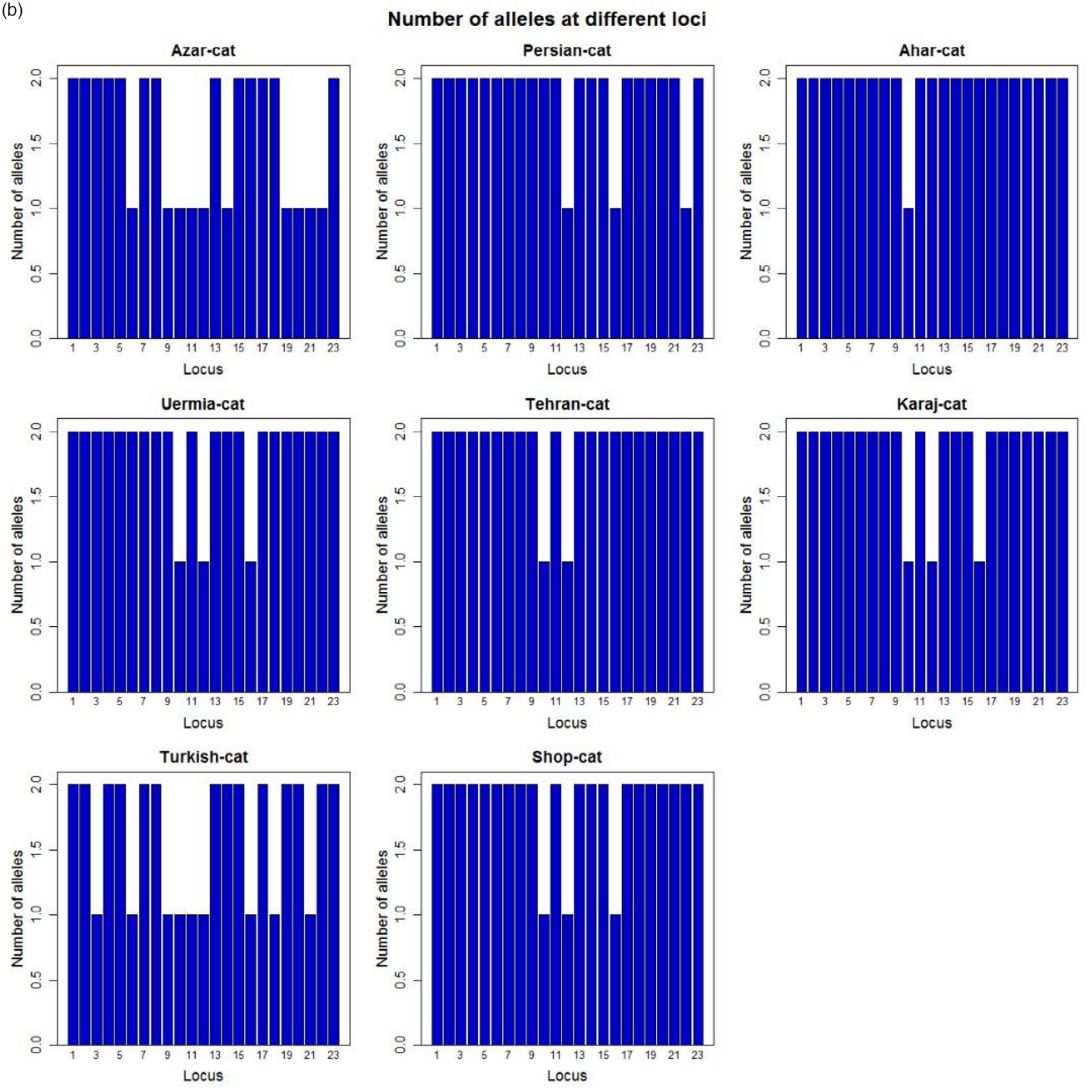

The expected and observed heterozygosity ranges across all loci were 0.34–0.75, with a mean of 0.57 and 1–0.33, respectively. According to this index, the populations of Azerbaijan and Turkish had the highest and lowest rates of heterozygosity, respectively. Furthermore, the highest and lowest heterozygosity rates were predicted for the populations from Azerbaijan and Uermia, respectively. Positions FCA77 and FCA35 in the Turkish population had the lowest observed heterozygosity, whereas positions FCA096 had the highest observed heterozygosity in all populations. The locus of FCa8 in the population had the highest expected heterozygosity. The role of FCA77 in the Azerbaijan population had this index's last and lowest value.

### 3.2 Inbreeding index

**FIGURE 3:**
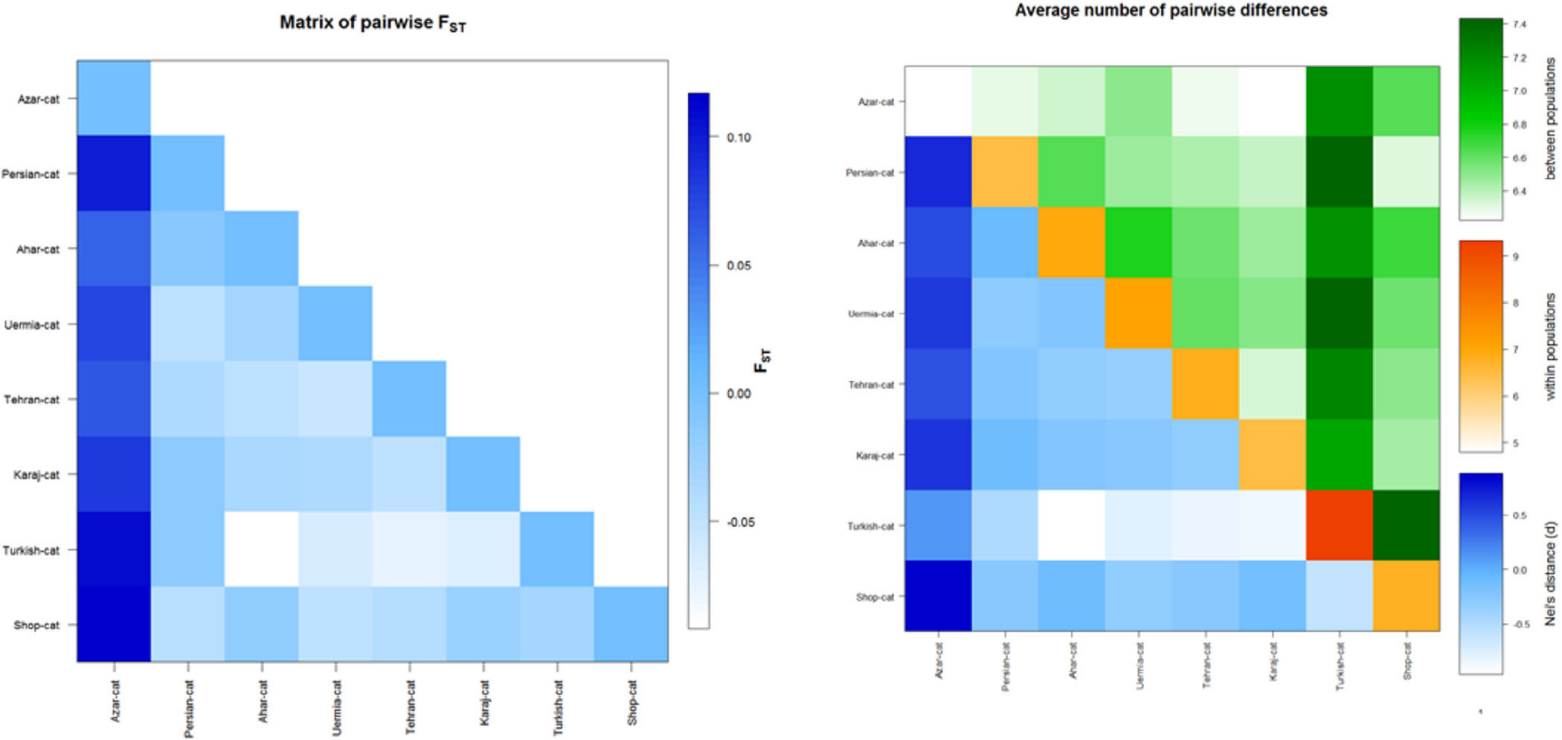
The degree of genetic differentiation between populations based on the *F*_ST_ index.

All loci have an *F*_st_ index range of 0.0190–0190.056, with an average of 0.03 for all loci, indicating low average genetic differentiation, and an average gene flow (*N*_m_) of 9.3 for all loci, which indicates high gene flow between samples. FCa096 and FCa35 had the highest gene flow, whereas FCa8 had the lowest. The FCa8 and FCa35 loci had the highest and lowest *F*_st_ indices in all populations, whereas the FCa8 and FCa096 loci had the highest and lowest inbreeding indices. According to this study, FCa77 was less successful in detecting differences between individuals than the marker Fca096, which had a higher Shannon index. The population of Azerbaijan has the highest genetic differentiation compared to the populations of Turkey, Uremia and Persian, as shown by the results of genetic differentiation based on the *F*_st_ index between populations based on ARLEQUIN software version 3.5. Figure 3 shows that the blue represents the *F*_st_ index.

The genetic differentiation of the population decreases as the colour increases and vice versa. Therefore, there is a high level of genetic differentiation and high genetic differentiation between the populations of Azerbaijan and Turkey, Uermia and Persia. Due to the low abundance of Ahar and Turkish cats, these two populations exhibit greater genetic mixing and gene flow. Furthermore, the Ahar population has minimal genetic differences from the Tehran and Karaj populations.

### 3.3 Genetic distance between studied population

The genetic similarity of the population decreases as the colour increases and vice versa. Therefore, there is a high level of genetic differentiation and high genetic differentiation between Azerbaijan and Turkey, Uermia and Persia populations. Due to the low abundances of Ahar and Turkey, these two populations exhibit greater genetic mixing and gene flow. Furthermore, the Ahar population has minimal genetic differences from the Tehran and Karaj populations. Turkish cats, Uermia and Persian populations showed the greatest genetic distance from Azerbaijan, whereas the populations of Ahar and Turkish cats showed the smallest genetic distance.

For this reason, there is a high gene flow between the Turkish and Ahar populations. This increases the level of heterozygosity in the population while reducing the likelihood of inbreeding and ultimately preventing problems associated with its after‐effects. In addition, it prevents the extinction of populations. In this way, it contributes to both population growth and conservation. Table 1 provided information about population difference on observed and expected allels and hetozygosity between studied cat population.

### 3.4 DAPC analysis

**FIGURE 4:**
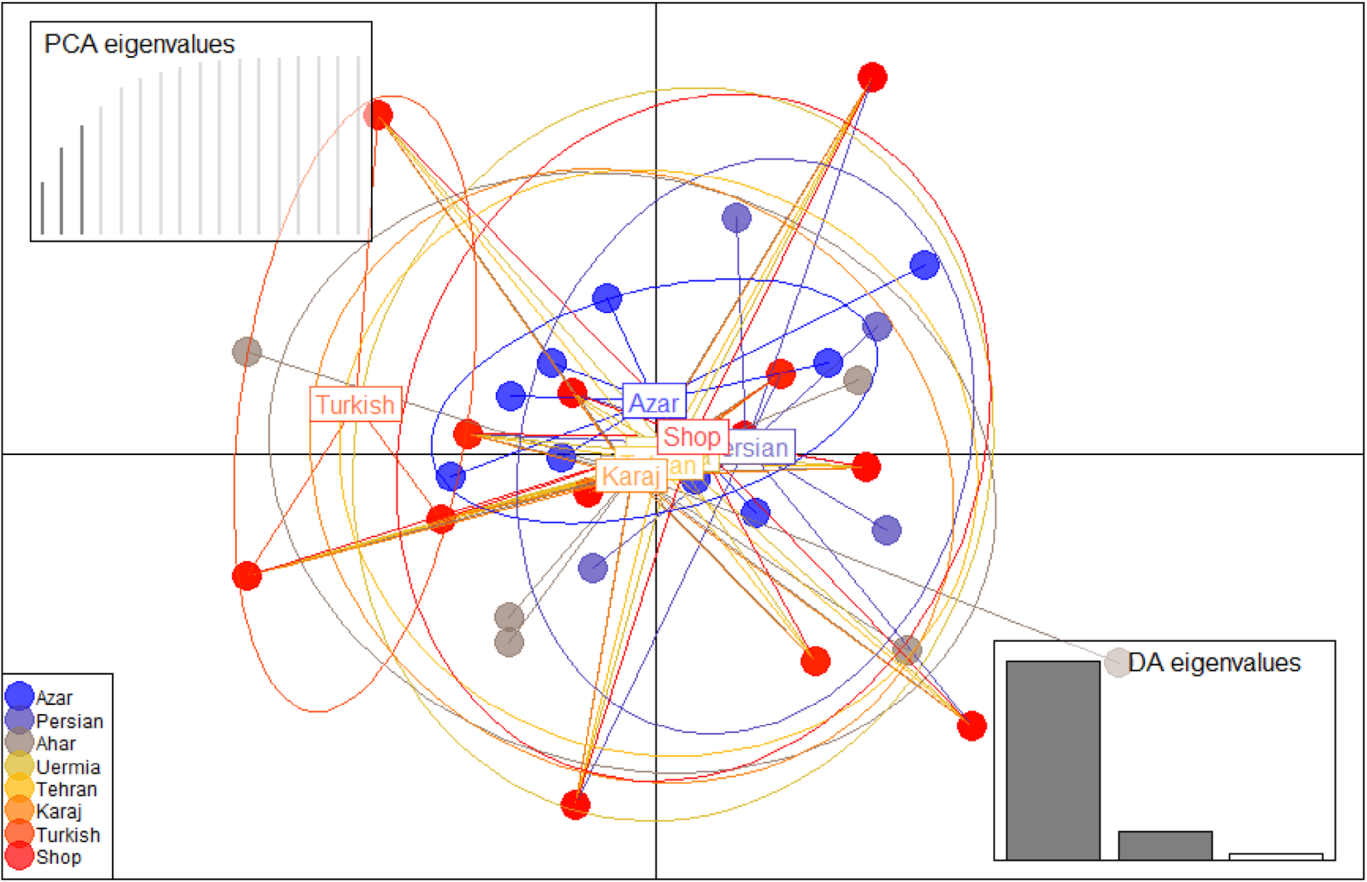
Principal component discriminant analysis (DAPC) analysis for the studiedcats based on the Adegenet package in the R software environment for each population.

In addition, pairwise discrimination within and between populations was examined using the genetic distance index. The results showed that the level of population differentiation is lower in the Azerbaijan population compared to other population groups and higher in the Turkish population. Within the population, the orange represents differentiation. In addition, the green hue indicates the population differences. The following figure illustrates the differences between Turkish‐Uermia, Turkish‐Persian and Turkish‐Shop. The Persian shop, Uermia‐Karaj, Tehran‐Karaj, Azerbaijan‐Karaj and Azerbaijan‐Tehran populations show minimal genetic differentiation. Principal component discriminant analysis (DAPC) analysis was performed using the Adegenet package in the R software environment to separate the studied samples (Figure 4). In this analysis, the geographically separated samples are displayed. According to the data, there is a high level of genetic mixing between populations. There may also be significant migration between populations.

**FIGURE 5:**
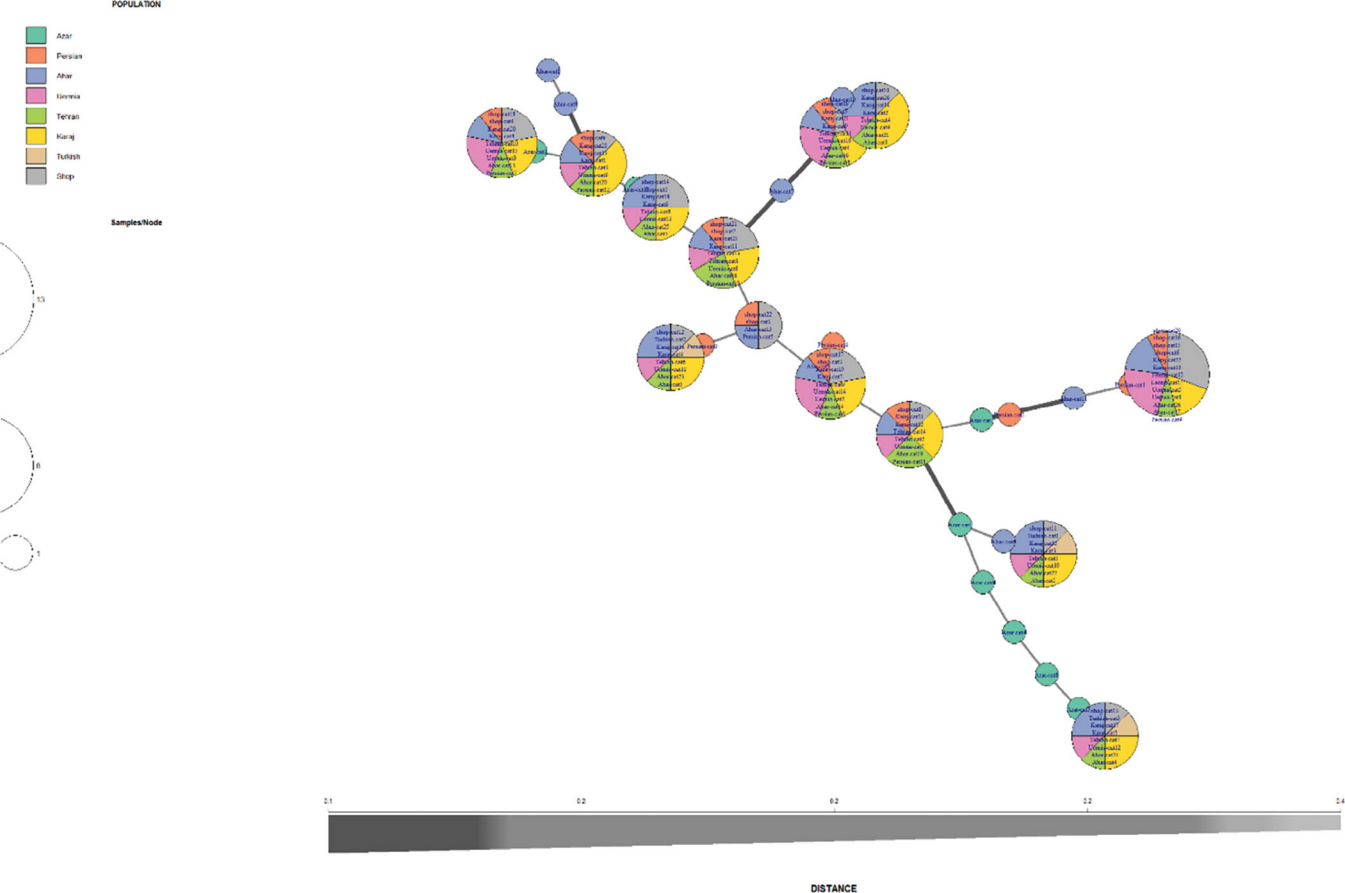
Genetic distance between individuals in the studied populations. Membership probability of each individual in each population based on principal component discriminant analysis (DAPC)‐based analysis.

Additionally, there is a significant migration rate between the Turkish and Ahar populations. Figure 5 illustrates the genetic distance between individuals in the studied populations. The membership probability of each individual in each population is based on DAPC‐based analysis.

### 3.5 STRUCTURE‐based analysis and admixture of alleles

**FIGURE 6:**
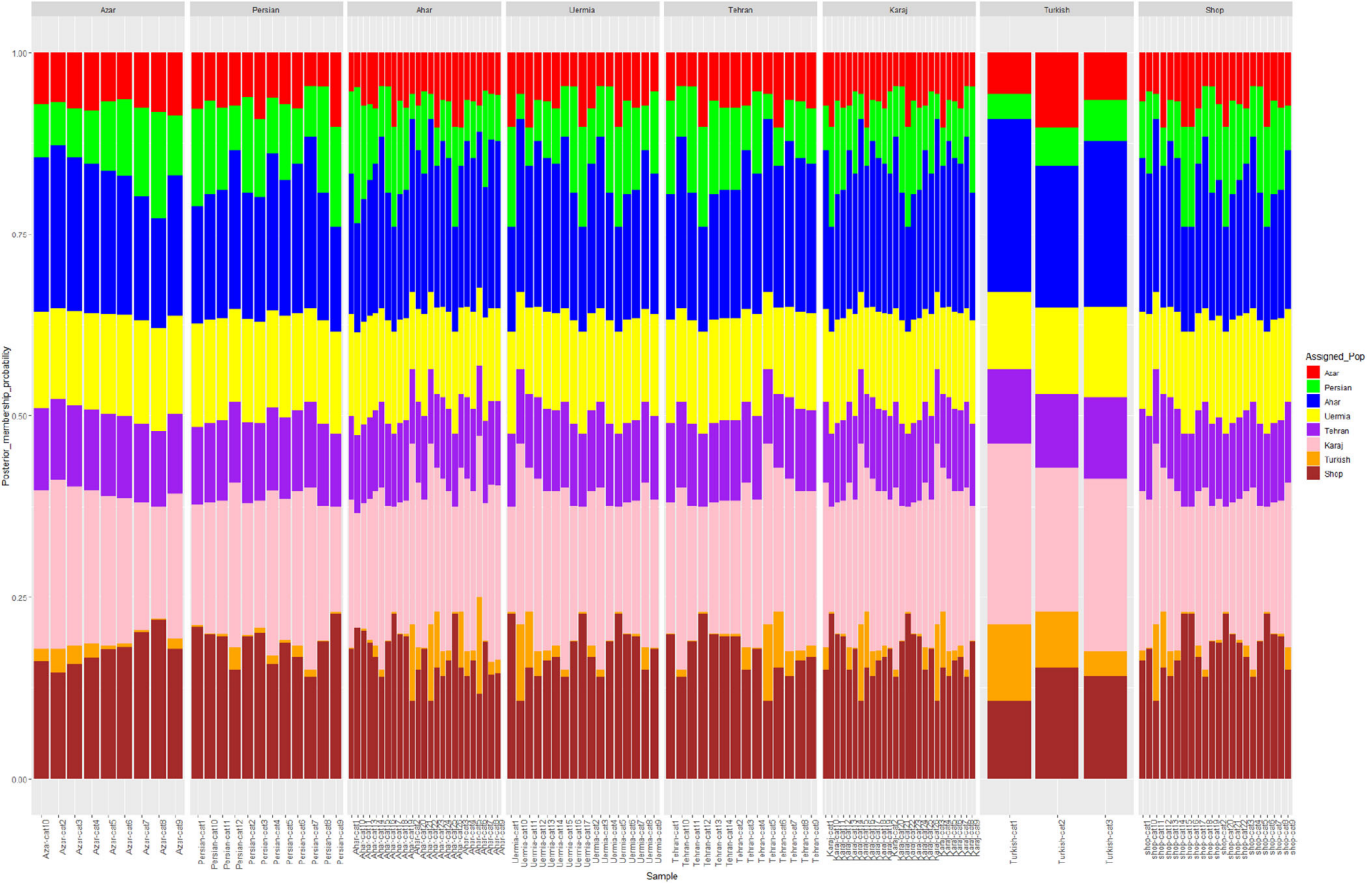
A graph for checking the membership probability of each person in each population.

**FIGURE 7:**
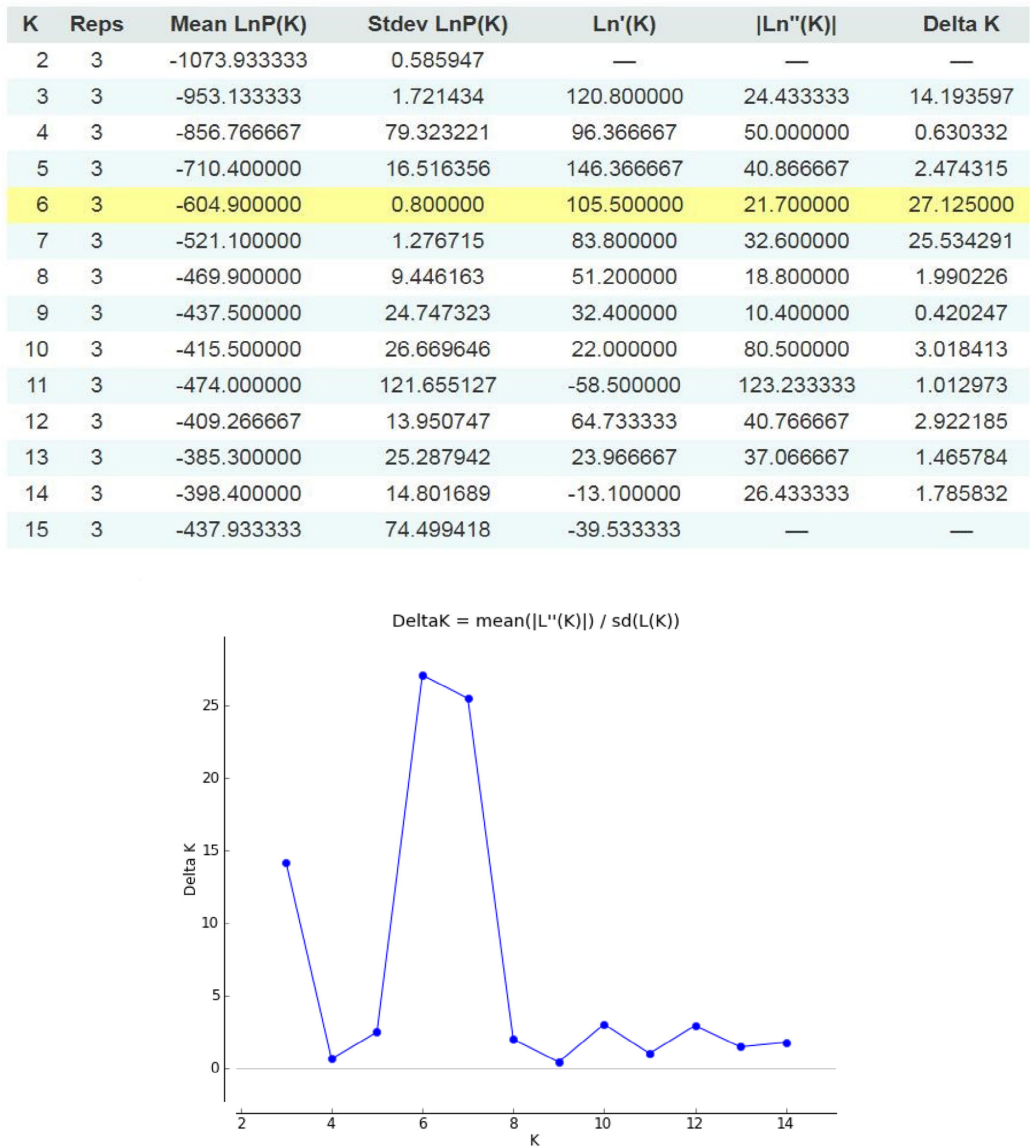
STRUCTURE‐based evidence outputs and allele admixture pattern and delta *K* value in studied cat populations.

In this study, the distance features of the populations were determined using structural software (Pritchard et al., 2000). The analysis showed that best is *K* = 6 shows the best delta *K* values. Consequently, six populations were assigned to the samples. Additionally, it provides the most accurate representation of genetic structure in this dataset. This is the basis for the stripes, which represent the percentage of each of the eight populations in different colours. Although genetic mixing between individuals was high, suggesting strong gene flow between individuals, the Bayesian clustering approach identified six distinct genetic clusters. The basis of this approach is the *K*‐statistic, which breaks the slope of the function at a point in the probability function where the hypothetical number *K* has the highest probability. Next, the maximum likelihood of the data (Ln*P* (*D*)) was calculated for each *K*. The number *K*, representing the number of populations, is more likely and accurate the smaller the amount of Ln*P* (*D*). Individuals with one particular colour are pure, as seen on the left, while individuals who work with different colours in different proportions are mixed and hybrid. The results suggest a high degree of genetic relatedness and admixture between individuals. Figure 6 addresses a graph for checking the membership probability of each person in each population. Figure 7 illustrates STRUCTURE‐based evidence outputs and allele admixture pattern in studied cat populations.

## 4 DISCUSSION

Maintaining genetic diversity is essential for the long‐term survival of most animal species. This conservation of genetic diversity should be based on a thorough knowledge of population structure, including the origins of each population's genetic variability. The interactions between a species and its environment can be influenced by variations in the genetic structure of a population, and populations with greater genetic diversity are better able to adapt to the risks posed by changing environmental conditions. Additionally, genetic diversity can reveal important details about a species’ current status, conservation efforts and evolutionary potential. It is widely accepted that microsatellites are practical molecular markers for assessing genetic divergence and diversity within and between populations. The study discovered notable polymorphisms and 112–226 range of the eight STR sizes. The FCa096 locus had the highest allelic diversity in the Persian population, whereas FCa35 and FCa77 had the lowest allelic diversity (Table 1). There were differences between populations, with an average of about 3.8 alleles per location. In contrast to the expected heterozygosity, which had a mean of 0.57 and ranged from 0.34 to 0.75, the observed heterozygosity had a mean of 0.62 and ranged from 1 to 0 (Table 2).

**TABLE 2:** Population differences based on observed and expected alleles and heterozygosity.

Pop		*N*	Na	Ne	I	Ho	He	uHe	*F*
**Azerbaijan**	Mean	9.000	3.200	2.155	0.869	0.689	0.500	0.529	−0.368
	SE	0.000	0.490	0.299	0.138	0.108	0.065	0.069	0.097
**Persian**	Mean	12.000	4.200	2.508	1.048	0.633	0.562	0.586	−0.130
	SE	0.000	1.020	0.411	0.196	0.097	0.063	0.066	0.098
**Ahar**	Mean	25.000	4.400	2.686	1.096	0.608	0.587	0.599	−0.014
	SE	0.000	1.122	0.432	0.193	0.111	0.064	0.065	0.097
**Uremia**	Mean	17.000	4.000	2.740	1.091	0.612	0.596	0.614	−0.016
	SE	0.000	0.894	0.433	0.189	0.105	0.063	0.065	0.095
**Tehran**	Mean	14.000	4.000	2.579	1.051	0.629	0.576	0.597	−0.085
	SE	0.000	0.894	0.388	0.174	0.099	0.061	0.063	0.097
**Karaj**	Mean	26.000	4.000	2.663	1.069	0.623	0.583	0.594	−0.057
	SE	0.000	0.894	0.436	0.183	0.104	0.065	0.066	0.089
**Turkish**	Mean	3.000	3.000	2.514	0.977	0.533	0.589	0.707	0.124
	SE	0.000	0.447	0.224	0.123	0.133	0.038	0.045	0.173
**Shop**	Mean	22.000	4.000	2.663	1.067	0.636	0.584	0.597	−0.079
	SE	0.000	0.894	0.415	0.187	0.102	0.067	0.068	0.072

*Note*: *F*, *F*‐statistics; He, expected heterozygosity; Ho, observed heterozygosity; I, Shannon index; *N*, number of samples; Na, observed allele; Ne, expected allele; uHe, mean of heterozygosity.

The *F*_st_ index showed that genetic differentiation was generally low, with the FCa8 locus showing the highest level of differentiation. High gene flow was detected between samples, with FCa096 and FCa35 having the highest. Of all populations, the Azerbaijan population had the highest level of genetic differentiation, whereas the Ahar and Tehran populations had the lowest level of genetic difference. The results showed gene flow's importance in preserving genetic diversity and preventing inbreeding. Additional evidence for genetic mixing and population migration was provided by analyses of genetic distance, pairwise differentiation and population structure. It is widely accepted that genetic variation within and between species populations can be measured using microsatellite markers. These markers have been used to identify geographically isolated populations and sibling species and are effective tools for identifying differences between closely related populations. Due to their high variability, ease of analysis and accuracy, microsatellites are the preferred markers for high‐resolution population analyses. According to studies comparing microsatellite variation and genome‐wide single nucleotide polymorphisms (SNPs) to identify potential biases, microsatellite allele richness is a better indicator of genome‐wide SNP diversity.

Furthermore, this study discovered that estimates of genetic differentiation between populations derived from microsatellite markers were significantly higher than estimates derived from SNPs, suggesting that marker selection needs to be carefully considered in population genetics studies (Fischer et al., 2017). Considering marker‐specific biases when interpreting genetic diversity estimates makes this finding relevant to the research. In addition, studies on the influence of the number of microsatellite markers on population genetic results have shown that the number of microsatellites examined influences the stability of genetic distances and population structures (Wang et al., 2021). This finding highlights the importance of considering the number of loci used for genotyping in microsatellite‐based population genetic studies. Driscoll et al. (2007) used microsatellite markers and mitochondrial sequencing to shed light on the origins of cat domestication. In this study, microsatellite markers were used to assess the origin of new varieties as they are a more reliable indicator of recent genetic diversity. The use of microsatellite markers in this study to clarify genetic diversity is consistent with these markers’ well‐recognized effectiveness and suitability in population genetics research. The results of this research contribute to our understanding of how microsatellite markers can be used to analyse genetic variation within and between domestic cat populations.

### 4.1 Allele frequencies within and between studied populations

Remarkably, our results showed that 23 alleles were identified in 128 samples. The highest number of alleles belonged to the FCa096 locus (eight alleles) in the Persian population, followed by FCa045 (seven alleles) in the Persian and Ahar populations. Another new finding is that the lowest number of alleles belonged to the FCa35 and FCa77 locus (two alleles) (Figure 2A). In this regard, comparing our research outputs, which are similar to the summary of previous literature, addresses some inconsistencies. For instance, Menotti‐Raymond and O'Brien (1995) using 10 microsatellite markers (FCA008, FCA023, FCA035, FCA043, FCA045, FCA077, FCA078, FCA090, FCA096 and FCA126) showed that they can amplify DNA samples from lions, cheetahs, pumas and Asian leopard cats. Furthermore, Hille et al. (2000) used a panel of eight feline microsatellite markers (F115, FCA031, FCA035, FCA096, FCA105, FCA124, FCA126 and FCA132) to study the genetic individualization of the European wild cat (*Felis silvestris*) and the domestic cat. Some possible explanations for the observed discrepancies include differences in sample size, population structure, breed history, specific SSR loci or genotyping techniques, or differences in the geographical distribution or variation in the number of SSRs applied for the measurement of genetic differentiation of cat samples. These results can be discussed in the context of other studies of genetic diversity in populations of randomly crossed cats and cat breeds.

A global study by examined the genetic distribution of domestic cats between breeds and randomly bred populations has shown that the genetic identities and historical data of purebred individuals provide insights into their origins. Using various breed assignment strategies and techniques, they examined cats from 29 premium breeds with SNPs and short tandem repeats. According to the study results, Paetkau's frequentist approach is better than Rannara and Mountain's Bayesian method at accurately assigning people to the appropriate breed. An additional study (Alhaddad 2013; Ito et al., 2020; Lyons 2013; Lyons et al., 2016) to genetically evaluate populations randomly crossed with breeds from around the world revealed a genetically diverse global cat population that could serve as a baseline gene pool for a given breed. We counted about 10 of them. The study also clarified the genetic characteristics of cats from the Mediterranean region, which includes Turkey, and how these characteristics differ from those of cats from other regions. Numerous studies, including Menotti‐Raymond et al. (2008), Lipinski et al. (2008), Pflueger et al. (2008), have provided insightful information about genetic diversity in populations of randomly bred cats and different cat breeds.

Provide information about population structure, genetic diversity and racial classification techniques. Using microsatellite markers, comparison and discussion of the results of these studies enable a comprehensive understanding of the genetic diversity between randomly bred domestic cats and Persian domestic cats. Phandee et al. (2016) used domestic cat (*F. catus*) microsatellite markers to measure genetic diversity in a group of 17 captive Asian golden cats (*Pardofelis temminckii, syn. captopuma* *temminckii*). A significant increase in the inbreeding coefficient was associated with a noticeable decrease in genetic diversity, as indicated by relatively lower observed heterozygosity than expected heterozygosity. These results highlight the importance of using microsatellite markers derived from domestic cats to study the genetic diversity of captive Asian golden cats and help develop successful breeding initiatives to conserve this species. Du et al. (2015) surveyed with the main aim of locating suitable microsatellite sites for genetic monitoring and elucidating the genetic makeup of the outbreeding population. Through their analysis, the researchers identified 31 polymorphic microsatellite markers, making them suitable for studying the genetic makeup of cat populations. In addition, the study mentioned above confirmed the classification of orange tabby cats as a well‐bred population. A thorough analysis of vital genetic parameters revealed that this specific cat population has a very high genetic diversity. It is important to emphasize that crucial variables, such as the number of loci examined, the heterozygosity detected at those loci and the sample size of animals included in each population, are necessary to estimate genetic diversity accurately (Barker, 1997).

### 4.2 Inbreeding index

The FCa8 locus showed the highest level of genetic differentiation, whereas the *F*_st_ index suggested a generally low level of differentiation (Figure 3). Gene flow analysis revealed high gene flow between samples, with the greatest gene flow observed in FCa096 and FCa35.Microsatellite markers were used in this study, and the results show high gene flow and low average genetic differentiation between samples (Figure 3). This is consistent with Lipinski et al. (2008), who determined the relationships between randomly bred cat populations and the pedigree lines of their offspring using a DNA marker panel using microsatellite markers. Consistent with the results of their study, they discovered a wide range of heterozygosity levels and inbreeding levels in cat breeds, indicating low genetic differentiation and high gene flow. Furthermore, the genetic differences between the populations in this study are consistent with the results of Kurushima et al. (2012), who examined the genetic differences between cat breeds and their populations of randomly bred individuals. They found that different cat populations had different levels of genetic differentiation, consistent with the finding that the Azerbaijan population has a higher level of genetic differentiation than the Turkish, Uremia and Persian cat populations. Furthermore, the observation that the Ahar population has minimal genetic differences from the Tehran and Karaj populations is consistent with the findings of Lipinski et al. (2008), who reported that most breeds descended from native cats of their supposed regions of origin, indicating minimal genetic differences between closely located populations. These findings could affect the conservation and management of different cat populations, expanding our knowledge of the genetic variability between them.

### 4.3 Genetic distance between studied population

The Ahar population had the lowest genetic difference from the Karaj and Tehran populations, whereas the Azerbaijan population had the highest genetic differentiation. The results showed that preventing inbreeding and maintaining genetic diversity depends on gene flow. Further evidence for genetic mixing and population migration has been provided by analyses of genetic distance, pairwise differentiation and population structure (Figure 4). The study's results are consistent with previous research on domestic cats’ genetic diversity and population composition. For example, Nilson et al. (2022) hypothesized that the Fertile Crescent was most likely the starting point for the domestication of cats, with the origins of these animals concentrated in the eastern Mediterranean basin, spreading to neighbouring islands, and eventually spreading south across the Levantine coast into the Nile Valley spread. This lends credence to the theory that populations in different geographic regions differ genetically. Furthermore, Randi et al. (2001) used mitochondrial DNA sequences and allelic variations at microsatellite loci to genotype wild and domestic cats in Italy. The study found that genetic variability was significantly distributed among the different groups, indicating different gene pools, which is consistent with the results of the current study. Furthermore, Lipinski et al. (2008) suggested that cat populations are more genetically isolated than human populations due to geographical location, which is consistent with the genetic differentiation observed in the current study between the Azerbaijan, Turkish, Uremia, Persian, Ahar, Tehran and Karaj populations. Anderson et al. (2022) genotyped 11,036 individuals (10,419 pedigree cats and 617 non‐pedigree cats) using commercial panel testing to determine the frequency and distribution of known genetic variants associated with diseases, blood types and physical traits in cat breeds. This is the largest DNA‐based study of domestic cats to date. The study highlighted the importance of thorough genetic screening across breeds by finding 13 disease‐associated variants in 47 breeds or breed types in which the variant had not previously been documented. The study concluded that the results support breeding programmes and the scientific community by providing valuable insights into cats’ genetic diversity and variant heritage. They also highlight the clinical utility of these data.

### 4.4 DAPC analysis

The results showed that the level of population differentiation is lower in the Azerbaijan population compared to other population groups and higher in the Turkish population. Within the population, the orange represents differentiation. In addition, the green hue indicates the population differences. The following figure illustrates the differences between Turkish‐U). DAPC analysis was performed based on the Adegenet package in the R software environment to separate the studied samples. In this analysis, the geographically separated samples are displayed. Urzi et al. (2021) conducted an in‐depth analysis using microsatellite markers to assess European wild cats’ genetic diversity and population structure. Researchers used DAPC and STRUCTURE analysis techniques to examine population structure to achieve this goal. These analysis techniques allowed the studied European wild cat populations to be divided into discrete genetic clusters and characterized. Using these cutting‐edge statistical methods, the study expanded our knowledge of the evolutionary dynamics of European wild cats. It guided conservation efforts by shedding light on these animals’ genetic diversity and population structure.

### 4.5 STRUCTURE‐based analysis and admixture of alleles

Although the first person of the Azerbaijan population is genetically more similar to the Karaj population, it shares some features with the Shop and Ahar populations. Migration between populations could be the cause. In addition, the first individual of the Turkish population has more genetic similarities with the Karaj population. Other members of the Ahar population have been the subject of studies. The graph shows that the first individual is genetically more similar to the Karaj population. These results suggest that the studied species may have high gene flow in the studied area. Additionally, there is strong genetic mixing between individuals, with a larger percentage of these peoples’ genes coming from the Karaj population (Figure 6). Examined genetic variation in 12 autosomal microsatellites from 34 wild and 64 domestic cats collected throughout Portugal. The study focused on the molecular analysis of hybridization between wild and domestic cats (*F. silvestris*). They used the technique of applying Bayesian approaches to analyse the composition and structure of populations. The results did not observe significant genetic differences between northern, central and southern wildcats. Furthermore, the results of our study here are subject to the same limitations regarding sample size and distribution of cats and need background information on the pedigree or mating history of such study materials. Of course, the limitations of the current studies include using outdated genotyping tools and a small number of SSR markers due to budget constraints. Future investigations are necessary to validate the kinds of conclusions that can be drawn from this study.

## 5 CONCLUSION

The domestic cat (*F. catus*) is a newly evolved species in the family Felidae that has developed some great features among mammals. It is critical to conserve these species and prevent inbreeding from reducing their genetic diversity by understanding their genetic relationships and applying the information to breeding management. The population was highly diverse, making it an excellent choice for studying genetic diversity and inbreeding phenomena.

To our knowledge, the study highlights the importance of understanding the genetic diversity between randomly bred domestic different geographically cats and Persian domestic cats using microsatellite markers. Overall, the results showed that the degree of differentiation within the population is high in the Turkish population compared to other population groups and lower in the Azerbaijan population. In addition, DAPC‐based analysis based on the ADAGENET package shows the distribution of samples by geographical locations. Moreover, the results show that genetic mixing between populations is high. On this basis, randomly bred domestic cats certainly have a high level of diversity compared to Persian domestic cats. This is an interesting topic for future work.

## AUTHOR CONTRIBUTIONS

**Shirin Mahmoodi**: Data analysis and interpretation. **Arash Javanmard and Ali Hojabr Rajeoni**: Conceptualization and writing—original draft. **Mehrshad Zeinolabedini and Mohammad Hossein Banabazi**: Project administration and software.

## CONFLICT OF INTEREST STATEMENT

The authors declare no conflicts of interest.

### ETHICS STATEMENT

All participants have given their written and informed consent.

### PEER REVIEW

The peer review history for this article is available at https://publons.com/publon/10.1002/vms3.70004.

## Data Availability

The data that support the findings of this study are available from the corresponding author upon reasonable request.
